# Incidence and risk of remnant gastric cancer after gastrectomy for gastric cancer: a population-based study from the SEER database

**DOI:** 10.1186/s12876-024-03133-x

**Published:** 2024-01-16

**Authors:** Shangcheng Yan, Ming Cheng, Wei Peng, Tianhua Liu, Jingyu Zhang, Mengchao Sheng, Rui Ren, Qiang Chen, Wei Gong, Yongyou Wu

**Affiliations:** 1 Department of Gastrointestinal Surgery, Second Affiliated Hospital of Soochow University, Suzhou, China; 2 Department of Gastroenterology and Minimally Invasive Surgery, Juntendo University Hospital, Tokyo, 113- 8431 Japan

**Keywords:** Gastric remnant, Second primary, Metachronous, Gastric cancer, Cumulative incidence

## Abstract

**Background:**

Gastric cancer (GC) constitutes a major global health problem, of which remnant gastric cancer (RGC) occurs less frequently. The rate of RGCs after gastrectomy for GC is increasing recently due to improved survival and screening, however, their incidence and risk have not been reported in the U.S. population. The objective of this study was to evaluate the incidence and elevated risk of RGC after GC gastrectomy in this population, and to identify the risk factors.

**Methods:**

Patients underwent gastrectomy for first primary GC in 2000–2015 and those who developed RGC were identified from Surveillance, Epidemiology and End Results (SEER) database. Fine-Gray regression was used to estimate the cumulative incidence and to identify risk factors. Standardized incidence ratios (SIRs) were calculated by Poisson regression to compare the risk with the general population.

**Results:**

Among 21,566 patients included in the cohort, 227 developed RGC. The 20-year cumulative incidence of RGC was 1.88%. Multivariate analysis revealed that older age, invasion depth, male sex, marital status, and lower income are independent risk factors for RGC development. SIR was 7.70 overall and > 4.5 in each stratum.

**Conclusions:**

Cumulative incidence and risk for RGCs increased continuously in patients underwent GC gastrectomy. Close and lifelong endoscopy surveillance should be recommended for patients who received GC gastrectomy, especially those with high-risk factors.

**Supplementary Information:**

The online version contains supplementary material available at 10.1186/s12876-024-03133-x.

## Background

Gastric cancer (GC) is the fifth most frequently diagnosed cancer and the fourth leading cause of cancer deaths worldwide [1]. Despite the declining and relatively low incidence in the U.S., GC still constitutes a major health problem given the poor survival and increasing incidence of early-onset cases [2]. Remnant gastric cancer (RGC), also known as gastric stump cancer, is a relatively rare entity occurring in the gastric remnant after previous partial gastrectomy for benign or malignant diseases [3]. Although RGC was initially referred only to RGC following benign conditions, its frequency has decreased due to improvement in anti-ulcer medications [4, 5]. On the other hand, the rate of RGC after gastrectomy for GC increased because of prolonged survival, improved screening, and increased function-preserving gastrectomy [5].

Patients subjected to gastrectomy have higher incidence and increased risk of RGC development than the general population, but the values vary greatly among studies with the incidence from almost zero to 7% and the increased risk of 4- to 7-fold [3, 6, 7]. Furthermore, though earlier studies on RGCs after ulcer gastrectomy came from Western countries, most recent researches on RGCs after GC gastrectomy were conducted in Asian populations [7, 8]. To the best of our knowledge, incidence and risk of RGC after gastrectomy for GC have not been reported in the U.S. population.

Moreover, several mechanisms have been found to explain the pathogenesis of RGC, including enterogastric reflux, *Helicobacter pylori* (*H. pylori*) infection, altered neurohormonal regulation, and molecular changes [5, 8]. However, very few studies analyzed the risk factors for RGC after GC gastrectomy, and none of them were performed in the U.S. population [9–13].

With this study, we intended to evaluate the incidence and elevated risk of RGC after GC gastrectomy in the U.S. using data from Surveillance, Epidemiology and End Results (SEER) program, and to identify the risk factors for RGC in this population.

## Methods

### Database and patients

**Fig. 1:**
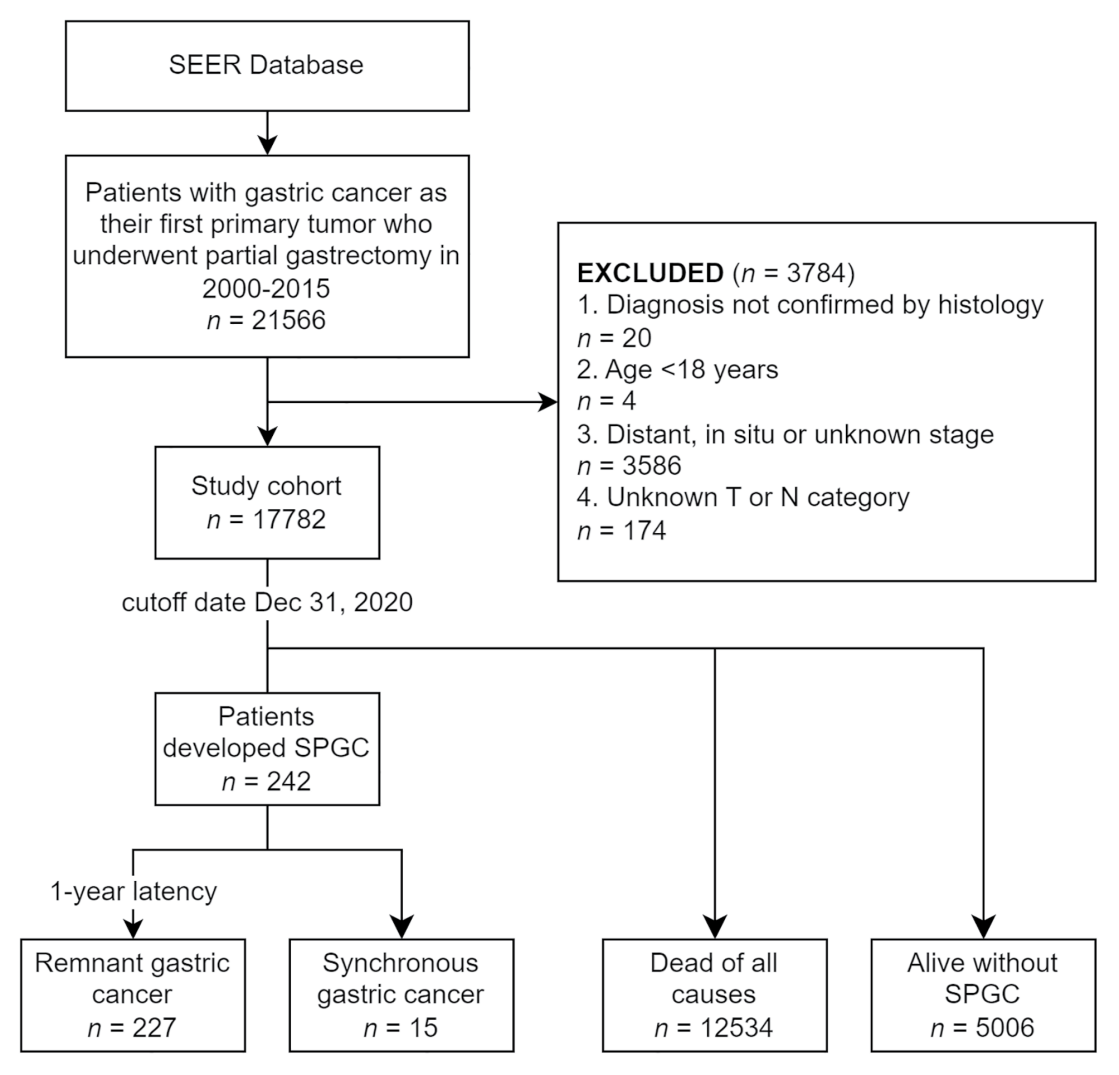
Flowchart of patient selection. *SEER* Surveillance, Epidemiology, and End Results; *SPGC* second primary gastric cancer

This is a population-based retrospective cohort study using data from National Cancer Institute’s SEER program. Ethics approval and participant consent were not necessary as this study involved the use of the previously-published de-identified data. Patient selection of this study is shown in Fig. 1.

Patients diagnosed with GC as their first primary tumor (first primary gastric cancer, FPGC) between January 1, 2000 and December 31, 2015 were identified in SEER database from 17 registries (covering 26.5% of U.S. total population). The cutoff date of this database was December 31, 2020. Identification of patients did not extend beyond 2015 to obtain a longer follow-up. Patients reported in Alaska or without county information were not identified because multiple primary standardized incidence ratio (MP-SIR) session in SEER*Stat excluded Alaska registries.

Patients with gastric (International Classification of Diseases for Oncology, 3rd Edition [ICD-O-3] topography codes C16.0-16.9) cancer (gastric epithelial tumors and poorly differentiated endocrine carcinoma, see Supplementary Table S1) who underwent partial gastrectomy (SEER codes 30–33, 41, 51, and 61, see Supplementary Table S2) were eligible for the study. Patients who received local excision (e.g. endoscopic submucosal dissection or mucosal resection) were not retrieved. The excluding criteria were: (1) diagnosis not confirmed by histology; (2) < 18 years of age at diagnosis; (3) tumor of distant, in situ or unknown stage; and (4) unknown T or N category.

### Definition and follow-up of RGC

An RGC was defined as a metachronous second primary GC (SPGC) following all types of partial gastrectomy for GC. SPGCs were indicated in the SEER database using “Sequence Number”, reported according to SEER rules for multiple primaries (MPs) which allows for the counting of new primary tumors at different subsites of the same organ. SPGCs diagnosed at least 1 year after FPGCs diagnosis were considered metachronous, otherwise synchronous [9, 12, 13]. Each patient was followed up from FPGC diagnosis until RGC diagnosis, diagnosis of synchronous SPGC, all-cause death, last follow-up, or December 31, 2020 (5 year after the last FPGC diagnosis), whichever came first.

### Study variables

Information on year of diagnosis, age at diagnosis, sex, race, marital status, area, income, anatomic site, tumor stage, TNM categories, tumor size, grade, histologic type, and surgical mode of FPGC, along with latency between FPGC and RGC, were collected from SEER database. Age was assessed in five categories (< 45, 45–54, 55–64, 65–74, and 75 + years), year of diagnosis was assessed in three categories (2000–2004, 2005–2009, and 2010–2015), and latency was assessed in three categories (12–59, 60–119, and 120 + months). Race was analyzed in five groups: non-Hispanic (NH) White; NH Black; NH Asian, including Asian and Pacific Islander; Hispanic; and NH others, including American Indian/Alaska Native and unknown race. Area was classified as urban and rural based on rural-urban continuum codes.

Anatomic site was divided into cardia (ICD-O-3 code C16.0), middle (C16.1, C16.2, C16.5, and C16.6), distal (C16.3 and C16.4), and not otherwise specified (NOS, C16.8 and C16.9). Tumor size was categorized as < 5 cm, 5 + cm and unknown. Tumor stage was classified as localized and regional according to SEER Stage (see Supplementary Table S3). TNM categories and histologic grade were redefined based on American Joint Committee on Cancer (AJCC) Staging Manual, 8th edition. Histologic type was characterized as intestinal (ICD-O-3 histologic codes 8010, 8140, 8211 and 8144), diffuse (8142, 8145 and 8490), and otherwise mixed/others, as defined by Lauren et al. [14]. Surgical mode was categorized into proximal gastrectomy (PG, code 33), distal gastrectomy (DG, code 31–32) and other partial gastrectomy (code 30, 41, 51, 61) based on SEER site-specific surgery codes (Supplementary Table S2).

### Statistical analysis

Study variables were summarized in the total cohort and compared between patients who developed RGC with who did not. Non-normally distributed continuous variables were presented as median and interquartile range (IQR) and compared with Mann-Whitney tests. Categorical variables were presented as number (%) and compared with *Chi*-square tests.

Fine-Gray competing risk regression analysis was conducted to calculate the cumulative incidence and 95% confidence interval (CI) of RGCs using “cuminc” function in R package “cmprsk”. Experiencing a synchronous SPGC or dying of any cause were considered competing events. Then, we performed multivariable analysis to estimate the adjusted subdistribution hazard ratios (HRs) and 95% CIs for RGC development using “crr” function according to Scrucca et al.’s method [15].

Standardized incidence ratios (SIRs) and 95% CIs were calculated via Poisson regression analysis in MP-SIR session of SEER*Stat. Poisson analyses were conducted in total cohort and stratified by study variables except for area and income (because their analyses were unavailable). SIR was defined as observed number of RGCs divided by expected number of GCs in the standardized U.S. general population. We also evaluated the dynamic SIRs overall and for each sex group with stratification by age at FPGC diagnosis, year of FPGC diagnosis and latency from FPGC diagnosis.

Data extraction was performed in case listing session of the SEER*Stat (version 8.4.1.1, Surveillance Research Program, National Cancer Institute, Calverton, MD, USA), while SIR analyses were completed in MP-SIR session with referent rates created in rate session. All other analyses were performed with R (version 4.2.1, R Core Team, Vienna, Austria). A two-sided *p* value < 0.05 was considered statistically significant.

## Results

### Patient characteristics

In total, 21,566 patients with FPGC underwent partial gastrectomy in 2000–2015 (Fig. 1). After exclusion, 17,782 patients were included in the final study cohort, of which 10,848 (61.0%) were male, 8197 (46.1%) were NH White, and the median age was 69 (IQR 59–77) years (Table 1). Distal (6837, 38.4%), G3 (10,866, 61.1%), and intestinal-type (12,216, 68.7%) GCs were the most prevalent in the total cohort.

**Table 1:** Baseline characteristics of patients in the study cohort at FPGC diagnosis

Characteristics	All patients	Development of RGC		
		No	Yes	*p*
Patients, n	17,782	17,555	227	
Year of diagnosis, median (IQR)	2007 (2003–2011)	2007 (2003–2011)	2007 (2004–2011)	0.799
Year group, n (%)				0.264
2000–2004	6070 (34.1)	6000 (34.2)	70 (30.8)	
2005–2009	5702 (32.1)	5618 (32.0)	84 (37.0)	
2010–2015	6010 (33.8)	5937 (33.8)	73 (32.2)	
Age at diagnosis (years), median (IQR)	69 (59–77)	69 (59–77)	66 (55–74)	< 0.001
Age group, n (%)				0.024
< 45 years	993 (5.6)	974 (5.5)	19 (8.4)	
45–54 years	2110 (11.9)	2075 (11.8)	35 (15.4)	
55–64 years	3642 (20.5)	3594 (20.5)	48 (21.1)	
65–74 years	5183 (29.1)	5113 (29.1)	70 (30.8)	
75 + years	5854 (32.9)	5799 (33.0)	55 (24.2)	
Sex, n (%)				0.410
Male	10,848 (61.0)	10,703 (61.0)	145 (63.9)	
Female	6934 (39.0)	6852 (39.0)	82 (36.1)	
Race, n (%)				0.446
NH White	8197 (46.1)	8102 (46.2)	95 (41.9)	
NH Black	2291 (12.9)	2264 (12.9)	27 (11.9)	
NH Asian	3905 (22.0)	3844 (21.9)	61 (26.9)	
Hispanic	3265 (18.4)	3223 (18.4)	42 (18.5)	
NH Others	124 (0.7)	122 (0.7)	2 (0.9)	
Marital status, n (%)				0.028
Married	10,779 (60.6)	10,620 (60.5)	159 (70.0)	
Widowed	2811 (15.8)	2784 (15.9)	27 (11.9)	
Single	2103 (11.8)	2077 (11.8)	26 (11.5)	
Divorced	1313 (7.4)	1303 (7.4)	10 (4.4)	
Other/Unknown	776 (4.4)	771 (4.4)	5 (2.2)	
Area, n (%)				1.000
Urban	16,253 (91.4)	16,046 (91.4)	207 (91.2)	
Rural	1529 (8.6)	1509 (8.6)	20 (8.8)	
Income, n (%)				0.233
<$55,000	2022 (11.4)	2004 (11.4)	18 (7.9)	
$55,000–64,999	2657 (14.9)	2624 (14.9)	33 (14.5)	
$65,000–74,999	5619 (31.6)	5536 (31.5)	83 (36.6)	
$75,000+	7484 (42.1)	7391 (42.1)	93 (41.0)	
Tumor site, n (%)				0.319
Cardia	3763 (21.2)	3720 (21.2)	43 (18.9)	
Middle	4910 (27.6)	4835 (27.5)	75 (33.0)	
Distal	6837 (38.4)	6754 (38.5)	83 (36.6)	
NOS	2272 (12.8)	2246 (12.8)	26 (11.5)	
Tumor stage, n (%)				0.064
Localized	7427 (41.8)	7318 (41.7)	109 (48.0)	
Regional	10,355 (58.2)	10,237 (58.3)	118 (52.0)	
T Category, n (%)				0.385
T1	4878 (27.4)	4817 (27.4)	61 (26.9)	
T2	2507 (14.1)	2466 (14.0)	41 (18.1)	
T3	6391 (35.9)	6314 (36.0)	77 (33.9)	
T4	4006 (22.5)	3958 (22.5)	48 (21.1)	
N Category, n (%)				0.016
N0	8338 (46.9)	8213 (46.8)	125 (55.1)	
N+	9444 (53.1)	9342 (53.2)	102 (44.9)	
Size, n (%)				0.442
< 5 cm	9688 (54.5)	9555 (54.4)	133 (58.6)	
5 + cm	5926 (33.3)	5856 (33.4)	70 (30.8)	
Unknown	2168 (12.2)	2144 (12.2)	24 (10.6)	
Grade, n (%)				0.787
G1	1059 (6.0)	1046 (6.0)	13 (5.7)	
G2	4975 (28.0)	4916 (28.0)	59 (26.0)	
G3	10,866 (61.1)	10,725 (61.1)	141 (62.1)	
Unknown	882 (5.0)	868 (4.9)	14 (6.2)	
Lauren classification, n (%)				0.791
Intestinal	12,216 (68.7)	12,057 (68.7)	159 (70.0)	
Diffuse	4246 (23.9)	4196 (23.9)	50 (22.0)	
Mixed/Others	1320 (7.4)	1302 (7.4)	18 (7.9)	
Surgical mode, n (%)				0.025
DG	6027 (33.9)	5946 (33.9)	81 (35.7)	
PG	1221 (6.9)	1196 (6.8)	25 (11.0)	
Others	10,534 (59.2)	10,413 (59.3)	121 (53.3)	

*FPGC* first primary gastric cancer; *RGC* remnant gastric cancer; *IQR* interquartile range; *NH* non-Hispanic; *NOS* not otherwise specified; *DG* distal gastrectomy; *PG* proximal gastrectomy

After 1-year latency, 227 patients (1.28%) developed RGC. The median latency between FPGCs and RGCs was 67 (IQR 42-112.5) months. There were no differences in most characteristics between patients with and without RGC, except for age, marital status, N category and surgical mode (Table 1).

### Cumulative incidence and risk factors of RGC

**Fig. 2:**
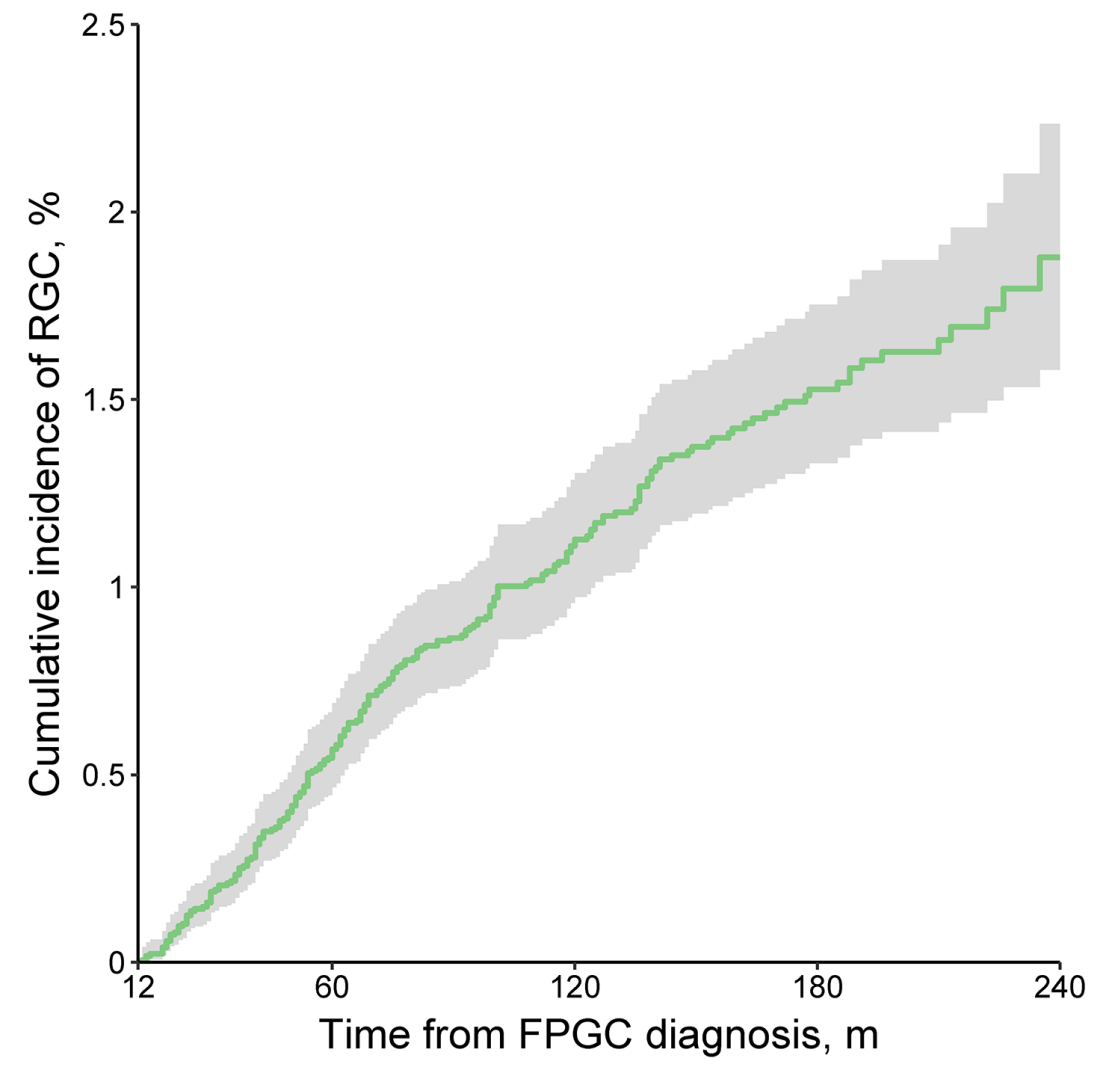
Cumulative incidence and 95% confidence interval of RGC after 1-year latency of FPGC diagnosis. *RGC* remnant gastric cancer; *FPGC* first primary gastric cancer

After 1-year latency from FPGC diagnosis, the cumulative incidence of RGC continued to increase over time without plateauing (Fig. 2). The cumulative incidence of RGC at 5 and 20 years after FPGC diagnosis was 0.57% (95% CI, 0.47–0.69%) and 1.88% (95% CI, 1.57–2.23%) respectively.

**Fig. 3:**
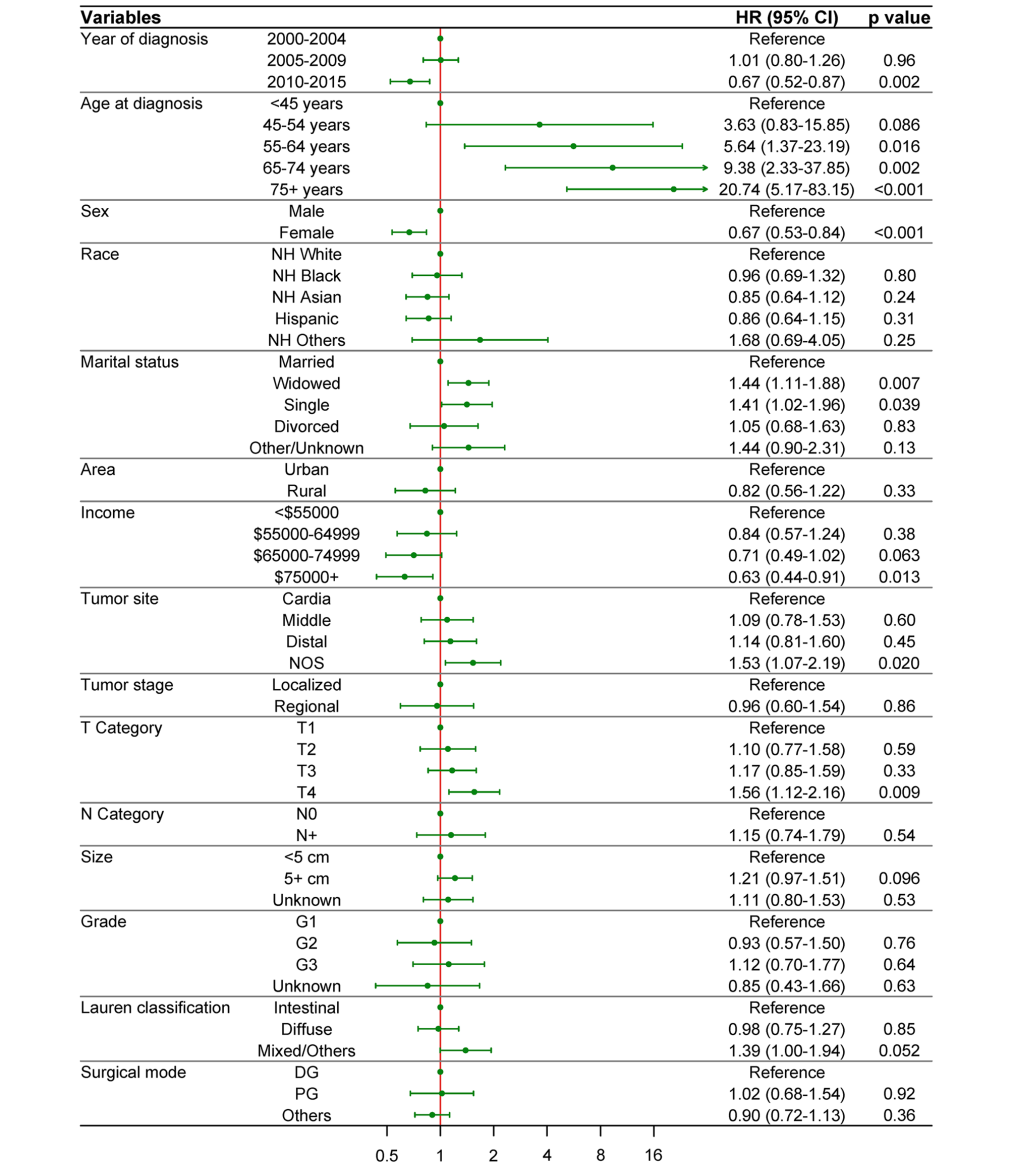
Adjusted subdistribution HR and 95% CI for RGCs based on multivariable competing risk analysis. *HR* hazard ratio; *CI* confidence interval; *RGCs* remnant gastric cancers; *NH* non-Hispanic; *NOS* not otherwise specified; *DG* distal gastrectomy; *PG* proximal gastrectomy

We then performed multivariate Fine-Gray competing risk regression analysis to identify the risk factors for RGC. The HR for RGC development increased with age and T category, and decreased with income (Fig. 3). Compared with young adults (< 45 years), patients aged 55–64 years (HR 5.64, 95% CI 1.37–23.19, *p* = 0.016), 65–74 years (HR 9.38, 95% CI 2.33–37.85, *p* = 0.002) and 75 + years (HR 20.74, 95% CI 5.17–83.15, *p* < 0.001) were at significantly higher risks to develop an RGC. Increased risks were also observed in widowed (HR 1.44, 95% CI 1.11–1.88, *p* = 0.007) or single patients (HR 1.41, 95% CI 1.02–1.96, *p* = 0.039), and patients with T4 tumor (HR 1.56, 95% CI 1.12–2.16, *p* = 0.009). Additionally, patients diagnosed in 2010–2015 had lower HR for RGC compared to those diagnosed earlier (0.67, 95% CI 0.52–0.87, *p* = 0.002), while NOS site tumors had higher HR than cardiac ones (HR 1.53, 95% CI 1.07–2.19, *p* = 0.020). On the other hand, female sex and income over $75,000 were associated with significantly lower risks of RGC development (HR 0.67, 95% CI 0.53–0.84, *p* < 0.001; HR 0.63, 95% CI 0.44–0.91, *p* = 0.013, respectively).

### Standardized incidence ratio of RGC

Overall GC risk after gastrectomy for FPGC was significantly higher than the U.S. general population (SIR 7.70, 95% CI 6.73–8.77, *p* < 0.05). Subgroup analyses showed that the elevated risk was consistent with statistically significant SIRs > 4.5 in each stratum (*p* < 0.05, Supplementary Table S4). Increase of SIRs were observed as stage, T and N category, size and grade of FPGCs increased. NH Asian patients had the lowest SIR (5.63, 95% CI 4.31–7.24) while Hispanic patients had the highest SIR (10.52, 95% CI 7.58–14.22) among all races. Concerning marital status, single patients were at the highest risk of RGCs compared with the general population (SIR 10.93, 95% CI 7.14–16.01). Across all tumor sites and surgical modes of FPGCs, cardia cancer and patients receiving PG had the highest SIR (8.82, 95% CI 6.38–11.88; 8.95, 95% CI 6.89–11.43, respectively).

**Fig. 4:**
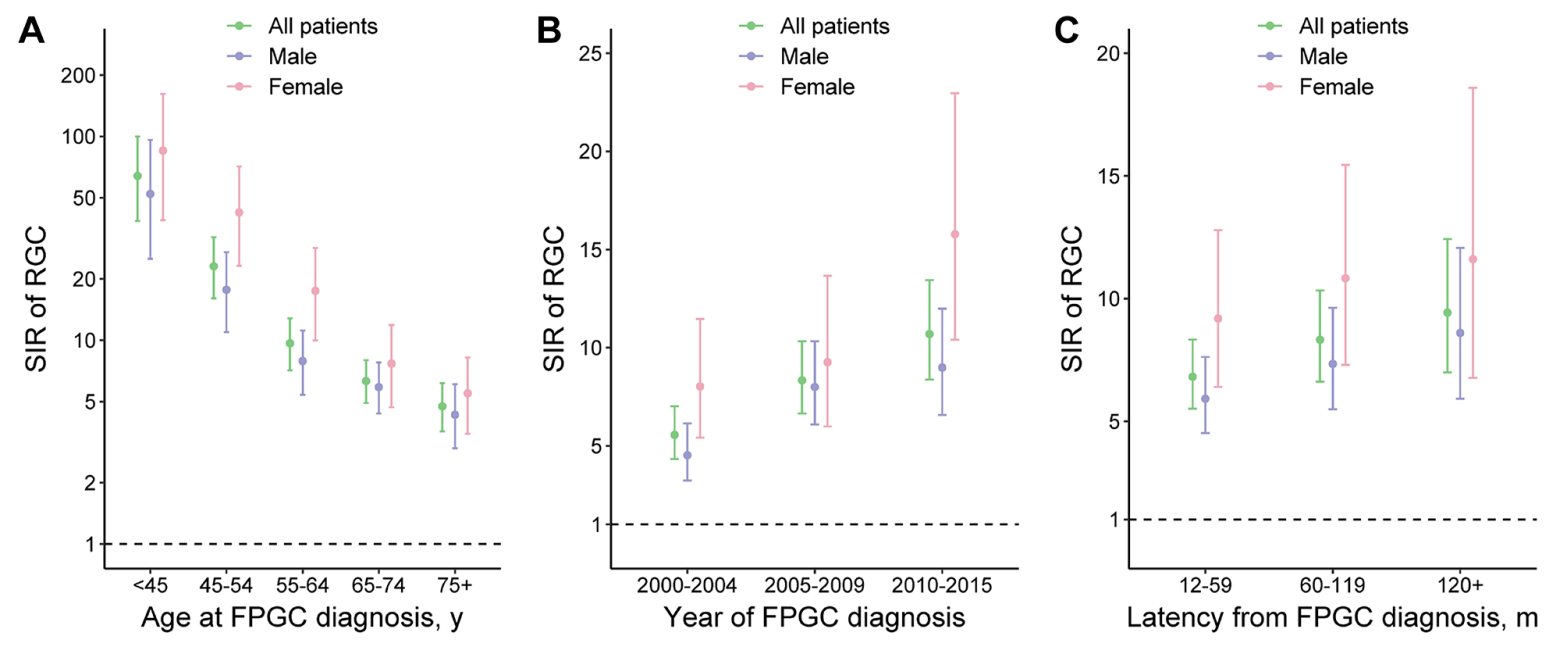
Dynamic SIRs for RGCs stratified by sex, age, year and latency. *SIRs* standardized incidence ratios; *RGCs* remnant gastric cancers; *FPGC* first primary gastric cancers

Additionally, we analyzed the dynamic SIRs for overall, male, and female patients (Supplementary Table S5). Female patients had the highest SIRs in all subgroups (Fig. 4). SIRs decreased with age at FPGC diagnosis (Fig. 4A), with the highest SIR observed in female patients under 45 years (85.12, 95% CI 38.92-161.59, *p* < 0.05). On the other hand, SIRs of RGC development increased together with year of FPGC diagnosis (Fig. 4B) and latency from FPGC diagnosis respectively (Fig. 4C).

## Discussion

This SEER-based study revealed that patients subjected to partial gastrectomy for GC had a 7.70-fold increased risk of developing GC than the U.S. general population and the 20-year cumulative incidence was 1.88%. As far as we know, this is the first U.S. population-based study on RGC following GC gastrectomy.

The incidence of RGC varies widely across previous studies (Table 2). A Japanese nationwide survey reported the precise incidence of metachronous RGC was 2.94% after 5-year follow-up [16], while a meta-analysis (mostly Asian studies) exclusively on RGCs after GC gastrectomy revealed a lower cumulative incidence of 1.2% [7]. Moreover, Mak et al. reported a pooled prevalence of 2.6% through meta-analysis, but the value is higher in European populations (5.8%) than that in Chinese populations (1.6%) [3]. In our study, we estimated the cumulative incidence of RGC after gastrectomy for GC in the U.S. population to be 0.57% at 5 years and 1.88% at 20 years after FPGC diagnosis. These discrepancies could be explained by several reasons. Different criterion was used to identify RGC by each study, while we followed the SEER criteria to define MPs which were considered more stringent [17, 18]. Moreover, a 1-year latency was used to exclude synchronous RGCs which might be missed at FPGC diagnosis. We also calculated the cumulative incidence with competing risk methods, which account for the high death rate of GC and produce lower estimates than traditional Kaplan-Meier methods [19]. Thus, we provided a reliable estimate of the incidence of RGC after gastrectomy for GC in the U.S. population, which was similar to that of the Asian populations.

**Table 2:** Prevalence of RGC after partial gastrectomy for GC

Study	Prevalence	Surgery	Design	Region
Kim 2014 [20]	0% ^a^	All	Single-center	Asia
Ryu 2016 [21]	0.7% ^a^	All	Single-center	Asia
Choi KS 2011 [22]	1.1% ^a^	All	Single-center	Asia
Choi Y 2021 [13]	1.1% ^a^	All	Single center	Asia
Ortigão 2022 [7]	CI: 1.2%	All	Meta-analysis	Europe & Asia
Jiang 2011 [23]	1.6% ^a^	PPG	Single-center	Asia
This study	20-year CI: 1.9%	All	Population	USA
Kinami 2021 [16]	2.1% ^a^	All	Population	Asia
Nakane 2021 [24]	5-year CI: 2.9%	All	Single-center	Asia
Morgagni 2014 [10]	20-year CI: 4.0%	DG	Single-center	Europe
Hanyu 2018 [25]	20-year CI: 5.4%	DG	Single-center	Asia
Aizawa 2020 [12]	10-year CI: 6.2%	PPG	Single-center	Asia
Ishida 2023 [26]	5-year CI: 5.7%	PG	Multicenter	Asia
Iwata 2018 [11]	5-year CI: 6.8%	PG	Single-center	Asia

^a^ Crude incidence. *RGC* remnant gastric cancer; *GC* gastric cancer; *CI* cumulative incidence; *DG* distal gastrectomy; *PG* proximal gastrectomy; *PPG* pylorus-preserving gastrectomy

Male sex, older age, depth of invasion, intestinal histologic type, and macroscopic type have been confirmed as independent risk factors for RGC after GC gastrectomy in previous studies [9–13], with which most of our findings were accordant. Intriguingly, we also identified widowed or single marital status, and lower income as independent risk factors (Fig. 3), which have not been reported before. Similar association was observed in the development of de novo GCs [27, 28]. Living with a partner improves overall well-being while widowed or single people might be at higher risk of bad lifestyle [29]. Similarly, lower income discourages the adoption of healthier lifestyle choices [30]. Patients with these factors are more likely to encounter carcinogens for GC such as tobacco and alcohol. This phenomenon may also be explained by that individuals with worse socioeconomic status have less chance to identify and treat precancerous lesions since they might be less adherent to GC surveillance [31]. Special attention should be directed to these vulnerable groups in post-gastrectomy management to prevent RGC.

PG and pylorus-preserving gastrectomy (PPG) are speculated to be risk factors for RGC because preserving the distal stomach or pylorus reduces bile reflux and increases the possibility of *H. pylori* infection [9, 11, 13]. Contrarily, we found no association between RGC development and tumor site or surgical mode. Although patients with tumor of NOS site had significantly higher risk (HR = 1.53, Fig. 3), it should not be considered as an independent risk factor given the uninformative nature. In fact, the role of bile reflux-*H. pylori* relation in RGC development remains at debate [5, 32]. Although Roux-en-Y reduces biliopancreatic reflux compared to Billroth-I reconstruction [33], the incidence of RGC following Roux-en-Y was reported significantly lower, questioning the hypothesis [16]. This inconsistency may also be attributed to SEER database’s incomplete information on tumor site and gastrectomy. In fact, large proportions of patients (59.2%) received unknown mode of partial gastrectomy in this study and specific type of reconstruction was not recorded in SEER database.

Another possible explanation could be the lower *H. pylori* infection rate in the U.S. than that in Asian countries, especially for urban areas where most of our cases (91.4%) lived [34]. Several factors contributed to this low prevalence, including strain difference [35], cost-effective vaccination [36], and high socioeconomic states [37]. A meta-analysis reported that *H. pylori* is prevalent in 35% U.S. population while the prevalence is over 50% in Japan, Korea and Italy, where most previous RGC studies took place [38]. Furthermore, this study included only patients of the last two decades, during which the infection rate decreased from 35.9 to 18.4% in the U.S [39]. Further studies are required to clarify the role of *H. pylori*, tumor site and surgical mode in RGC development.

We found that female patients were at significantly lower risk of developing RGC (HR = 0.67, Fig. 3), which is consistent with previous studies [9–11]. However, in comparison with the general population, female had higher SIR than male in all subgroups (Fig. 4), indicating a more prominent increased risk than male. This discrepancy might be owing to the higher incidence of de novo GC in male than that in female [1, 7, 40], potentially diluting the increased risk for RGC in male patients. Similar phenomenon has been observed in second primary lung cancer [41] and colorectal cancer [42], both known as male-predominant cancer.

It is intuitive that earlier year of diagnosis was associated with a significantly higher risk of RGC because the incidence and risk increased for each year (Figs. 2 and 4B). Nevertheless, our analysis also revealed that age and invasion depth increased the risk of RGC (Fig. 3). Older age has been previously reported as an independent risk factor for metachronous multiple cancers including RGC [9, 42, 43, 44], though this seemed counterintuitive because younger patients with FPGC have more time to develop RGC. Indeed, we did observe a higher SIR in younger patients in comparison with the general population (Fig. 4A). This phenomenon could be explained by the multicentric carcinogenesis theory of SPGCs [9, 45]. RGCs originate from noncancerous mucosa of the gastric remnant which is constantly influenced by the carcinogenic factors. The older the patient is, the longer time has passed to the progression of multicentric carcinogenesis. This time-dependent manner could also explain the increased RGC risk with deeper depth of invasion [6, 8, 9]. Also, the incidence of overall GC is higher in older people, which thus dilutes the SIR of RGC, similar to other second primary cancers [42, 43, 44].

In our analysis, patients underwent gastrectomy for GC were at significantly higher risk of developing RGC than the general population across all strata (SIR > 4.5, Supplementary Table S4). Taking together the time-dependently increasing SIR and cumulative incidence of RGC (Figs. 2 and 4C), our findings suggested that patients underwent gastrectomy for GC should receive lifelong endoscopic surveillance. Current guidelines require a follow-up for the first 5 years after surgery, but individualized follow-up plans beyond that period was also encouraged to detect RGCs [46]. Thus, risk factors found in our study could help tailoring surveillance strategies for individual patients.

There were some limitations to this study that should be considered when interpreting the results. Firstly, our analyses used the latencies from FPGC diagnosis instead of those from gastrectomy because the date of surgery is unknown in SEER database. Secondly, recurrences and metastases might be recorded mistakenly in the database, which could overestimate the rate of RGCs. However, this bias might be tiny because SEER follows criteria for primary tumors strictly [47]. Moreover, the generalizability of our study might be limited because SEER areas are more urban. This cohort had a higher proportion of T1 patients (27.4%), and a lower percentage of White patients (46.1%) than that of non-SEER areas [48]. Lastly, several important factors in GC carcinogenesis such as *H. pylori* infection, smoking, alcohol use, comorbidities, reconstruction methods etc., were not included in our analyses because SEER database does not collect these data. Given the difficulty to conduct a randomized controlled trial on RGC, multi-center studies using prospectively collected data from a finer scale would be needed in the future to compensate for the limitations of this database study.

## Conclusions

In conclusion, we found the cumulative incidence of RGCs was 1.88% at 20 years after GC gastrectomy in the U.S. population. Older age, male sex, marital status, lower income, and invasion depth are independent risk factors for RGC development. Patients underwent GC gastrectomy were at significant higher risk to develop GC than the U.S. general population. Close and lifelong endoscopy surveillance should be recommended for these patients, especially those accompanied by high-risk factors.

### Electronic supplementary material

Below is the link to the electronic supplementary material.

Supplementary Material 1

## Data Availability

The datasets used and analyzed during the current study are available at https://seer.cancer.gov/data/ or from the corresponding author on reasonable request.

## Abbreviations

AJCC: American Joint Committee on Cancer
CI: Confidence interval
DG: Distal gastrectomy
FPGC: First primary gastric cancer
GC: Gastric cancer
HR: Hazard ratio
H. pylori: Helicobacter pylori
ICD-O-3: International Classification of Diseases for Oncology, 3rd Edition
IQR: Interquartile range
MP: Multiple primary
MPGC: Multiple primary gastric cancer
NH: Non-Hispanic
NOS: Not otherwise specified
PG: Proximal gastrectomy
PPG: Pylorus-preserving gastrectomy
RGC: Remnant gastric cancer
SPGC: Second primary gastric cancer
SIR: Standardized incidence ratio
SEER: Surveillance, Epidemiology and End Results
