# Supplementary material for: Incidence and risk of remnant gastric cancer after gastrectomy for gastric cancer: a population-based study from the SEER database
Source: BMC Gastroenterol. 2024 Jan 16;24:35. doi: 10.1186/s12876-024-03133-x (PMC10790470; doi:10.1186/s12876-024-03133-x)
Supplement: Supplementary file 1 — Supplementary Material 1 [file 12876_2024_3133_MOESM1_ESM.docx]

**Supplementary Table S1** Definition of gastric cancer in this study by ICD-O-3 histology code

| **Tumor** | **ICD-O-3 Histology Code** |
| --- | --- |
| Epithelial Tumors of Stomach |  |
| Adenocarcinoma | 8050, 8140-8145, 8147, 8190, 8201, 8210-8211, 8214, 8221, 8230-8231, 8255, 8260-8263, 8290, 8310, 8315, 8320, 8323, 8333, 8380-8384, 8401, 8440-8441, 8450, 8480-8482, 8490, 8500, 8503-8504, 8510, 8512, 8514, 8525, 8542, 8550-8551, 8560, 8562, 8570-8576 |
| Squamous cell carcinoma | 8004, 8032, 8051-8052, 8070-8076, 8078, 8082-8084, 8980 |
| Salivary gland-type tumor | 8200, 8430 |
| Undifferentiated carcinoma | 8020-8022 |
| Other epithelial tumors | 8000-8001, 8003, 8005, 8010-8012, 8030-8031, 8033, 8035, 8040, 8122-8123, 8130, 8951 |
| Gastroenteropancreatic neuroendocrine tumors |  |
| Poorly differentiated endocrine carcinoma of pancreas and digestive tract | 8002, 8013, 8041-8045, 8246 |

*ICD-O-3* International Classification of Diseases for Oncology, 3^rd^ Edition.

**Supplementary Table S2** Surgery codes of gastrectomy in SEER Program Coding and Staging Manual 2023

| **Codes** | **SEER Description** | **Partial Gastrectomy** | **Surgical Mode** |
| --- | --- | --- | --- |
| 30 | Gastrectomy, NOS (partial, subtotal, hemi-) | Yes | Others |
| 31 | Antrectomy, lower (distal-less than 40% of stomach) | Yes | DG |
| 32 | Lower (distal) gastrectomy (partial, subtotal, hemi-) | Yes | DG |
| 33 | Upper (proximal) gastrectomy (partial, subtotal, hemi-) | Yes | PG |
| 40 | Near-total or total gastrectomy, NOS |  | NTG/TG |
| 41 | Near-total gastrectomy | Yes | Others |
| 42 | Total gastrectomy |  | TG |
| 50 | Gastrectomy, NOS WITH removal of a portion of esophagus |  | NOS |
| 51 | Partial or subtotal gastrectomy | Yes | Others |
| 52 | Near total or total gastrectomy |  | NTG/TG |
| 60 | Gastrectomy with a resection in continuity with the resection of other organs, NOS |  | NOS |
| 61 | Partial or subtotal gastrectomy, in continuity with the resection of other organs | Yes | Others |
| 62 | Near total or total gastrectomy, in continuity with the resection of other organs |  | NTG/TG |
| 63 | Radical gastrectomy, in continuity with the resection of other organs |  | NOS |
| 80 | Gastrectomy, NOS |  | NOS |

*SEER* Surveillance, Epidemiology, and End Results; *NOS* not otherwise specified; *DG* distal gastrectomy; *PG* proximal gastrectomy; *NTG* near total gastrectomy; *TG* total gastrectomy.

**Supplementary Table S3** Description of SEER Stage

| **SEER Stage** | **Description** |
| --- | --- |
| In situ | A noninvasive neoplasm; a tumor which has not penetrated the basement membrane nor extended beyond the epithelial tissue. Some synonyms are intraepithelial (confined to epithelial tissue), noninvasive and noninfiltrating. |
| Localized | An invasive neoplasm confined entirely to the organ of origin. It may include intraluminal extension where specified. For example for colon, intraluminal extension limited to immediately contiguous segments of the large bowel is localized, if no lymph nodes are involved. Localized may exclude invasion of the serosa because of the poor survival of the patient once the serosa is invaded. |
| Regional | A neoplasm that has extended 1) beyond the limits of the organ of origin directly into surrounding organs or tissues; 2) into regional lymph nodes by way of the lymphatic system; or 3) by a combination of extension and regional lymph nodes. |
| Distant | A neoplasm that has spread to parts of the body remote from the primary tumor either by direct extension or by discontinuous metastasis (e.g., implantation or seeding) to distant organs, issues, or via the lymphatic system to distant lymph nodes. |
| Unknown | Blank/Unstaged - Information is not sufficient to assign a stage. |

Over time several different Extent of Disease schemes have been used. *SEER* Surveillance, Epidemiology, and End Results.

**Supplementary Table S4** SIR analyses of RGCs following gastrectomy for FPGCs, stratified by FPGC characteristics

| **Characteristics** | **Observed, n** | **Expected, n** | **SIR (95% CI)** |
| --- | --- | --- | --- |
| All patients | 227 | 29.50 | 7.70 (6.73-8.77) |
| Latency, n (%) |  |  |  |
| 12-59 months | 95 | 14.34 | 6.62 (5.36-8.10) |
| 60-119 months | 82 | 9.85 | 8.32 (6.62-10.33) |
| 120+ months | 50 | 5.30 | 9.43 (7.00-12.43) |
| Year of diagnosis, n (%) |  |  |  |
| 2000-2004 | 70 | 12.60 | 5.56 (4.33-7.02) |
| 2005-2009 | 84 | 10.07 | 8.34 (6.65-10.33) |
| 2010-2015 | 73 | 6.83 | 10.69 (8.38-13.44) |
| Age at diagnosis, n (%) |  |  |  |
| <45 years | 19 | 0.30 | 63.95 (38.5-99.87) |
| 45-54 years | 35 | 1.52 | 23.07 (16.07-32.08) |
| 55-64 years | 48 | 4.97 | 9.66 (7.12-12.81) |
| 65-74 years | 70 | 11.10 | 6.31 (4.92-7.97) |
| 75+ years | 55 | 11.61 | 4.74 (3.57-6.16) |
| Sex, n (%) |  |  |  |
| Male | 145 | 21.35 | 6.79 (5.73-7.99) |
| Female | 82 | 8.15 | 10.06 (8.00-12.49) |
| Race, n (%) |  |  |  |
| NH White | 95 | 10.64 | 8.93 (7.22-10.91) |
| NH Black | 27 | 3.84 | 7.03 (4.63-10.23) |
| NH Asian | 61 | 10.83 | 5.63 (4.31-7.24) |
| Hispanic | 42 | 3.99 | 10.52 (7.58-14.22) |
| NH Others | 2 | 0.20 | 10.17 (1.23-36.72) |
| Marital status, n (%) |  |  |  |
| Married | 159 | 20.17 | 7.88 (6.71-9.21) |
| Widowed | 27 | 4.23 | 6.38 (4.21-9.29) |
| Single | 26 | 2.38 | 10.93 (7.14-16.01) |
| Divorced | 10 | 1.65 | 6.07 (2.91-11.15) |
| Other/Unknown | 5 | 1.07 | 4.69 (1.52-10.94) |
| Tumor site, n (%) |  |  |  |
| Cardia | 43 | 4.88 | 8.82 (6.38-11.88) |
| Middle | 75 | 9.12 | 8.22 (6.47-10.31) |
| Distal | 83 | 12.08 | 6.87 (5.47-8.52) |
| NOS | 26 | 3.42 | 7.61 (4.97-11.15) |
| Tumor stage, n (%) |  |  |  |
| Localized | 109 | 17.94 | 6.08 (4.99-7.33) |
| Regional | 118 | 11.55 | 10.21 (8.45-12.23) |
| T Category, n (%) |  |  |  |
| T1 | 61 | 12.78 | 4.77 (3.65-6.13) |
| T2 | 41 | 5.01 | 8.18 (5.87-11.1) |
| T3 | 77 | 8.32 | 9.26 (7.31-11.57) |
| T4 | 48 | 3.35 | 14.33 (10.56-18.99) |
| N Category, n (%) |  |  |  |
| N0 | 125 | 19.34 | 6.46 (5.38-7.70) |
| N+ | 102 | 10.16 | 10.04 (8.19-12.19) |
| Size, n (%) |  |  |  |
| <5 cm | 133 | 18.22 | 7.30 (6.11-8.65) |
| 5+ cm | 70 | 7.67 | 9.12 (7.11-11.53) |
| Unknown | 24 | 3.60 | 6.66 (4.27-9.91) |
| Grade, n (%) |  |  |  |
| G1 | 13 | 2.60 | 5.00 (2.66-8.54) |
| G2 | 59 | 10.36 | 5.70 (4.34-7.35) |
| G3 | 141 | 14.94 | 9.44 (7.95-11.13) |
| Unknown | 14 | 1.60 | 8.77 (4.79-14.71) |
| Lauren classification, n (%) |  |  |  |
| Intestinal | 159 | 22.35 | 7.11 (6.05-8.31) |
| Diffuse | 50 | 4.93 | 10.13 (7.52-13.36) |
| Mixed/Others | 18 | 2.21 | 8.13 (4.82-12.85) |
| Surgical mode, n (%) |  |  |  |
| DG | 81 | 11.69 | 6.93 (5.50-8.61) |
| PG | 25 | 1.89 | 13.23 (8.56-19.54) |
| Others | 121 | 15.91 | 7.60 (6.31-9.08) |

Standardized incidence ratios (SIRs) are defined as observed/expected numbers. P values are <0.05 in all strata. *RGCs* remnant gastric cancers; *FPGCs* first primary gastric cancers; *CI* confidence interval; *IQR* interquartile range; *NH* non-Hispanic; *NOS* not otherwise specified; *DG* distal gastrectomy; *PG* proximal gastrectomy.

**Supplementary Table S5** Dynamic SIRs of RGCs in overall cohort and each sex group with stratification for age at FPGC diagnosis, year of FPGC diagnosis and latency from FPGC diagnosis

| **Groups** | **Observed** | **Expected** | **O/E** | **CI Lower** | **CI Upper** |
| --- | --- | --- | --- | --- | --- |
| **All patients** |  |  |  |  |  |
| Age at diagnosis, years |  |  |  |  |  |
| <45 | 19 | 0.30 | **63.95** | 38.50 | 99.87 |
| 45-54 | 35 | 1.52 | **23.07** | 16.07 | 32.08 |
| 55-64 | 48 | 4.97 | **9.66** | 7.12 | 12.81 |
| 65-74 | 70 | 11.10 | **6.31** | 4.92 | 7.97 |
| 75+ | 55 | 11.61 | **4.74** | 3.57 | 6.16 |
| Year of diagnosis |  |  |  |  |  |
| 2000-2004 | 70 | 12.60 | **5.56** | 4.33 | 7.02 |
| 2005-2009 | 84 | 10.07 | **8.34** | 6.65 | 10.33 |
| 2010-2015 | 73 | 6.83 | **10.69** | 8.38 | 13.44 |
| Latency, months |  |  |  |  |  |
| 12-59 | 95 | 13.94 | **6.82** | 5.52 | 8.33 |
| 60-119 | 82 | 9.85 | **8.32** | 6.62 | 10.33 |
| 120+ | 50 | 5.30 | **9.43** | 7.00 | 12.43 |
| **Male** |  |  |  |  |  |
| Age at diagnosis, years |  |  |  |  |  |
| <45 | 10 | 0.19 | **52.26** | 25.06 | 96.10 |
| 45-54 | 21 | 1.19 | **17.69** | 10.95 | 27.04 |
| 55-64 | 32 | 4.05 | **7.90** | 5.40 | 11.15 |
| 65-74 | 50 | 8.49 | **5.89** | 4.37 | 7.76 |
| 75+ | 32 | 7.42 | **4.31** | 2.95 | 6.09 |
| Year of diagnosis |  |  |  |  |  |
| 2000-2004 | 40 | 8.86 | **4.52** | 3.23 | 6.15 |
| 2005-2009 | 59 | 7.37 | **8.00** | 6.09 | 10.33 |
| 2010-2015 | 46 | 5.12 | **8.99** | 6.58 | 11.99 |
| Latency, months |  |  |  |  |  |
| 12-59 | 60 | 10.13 | **5.92** | 4.52 | 7.62 |
| 60-119 | 52 | 7.08 | **7.34** | 5.49 | 9.63 |
| 120+ | 33 | 3.84 | **8.60** | 5.92 | 12.07 |
| **Female** |  |  |  |  |  |
| Age at diagnosis, years |  |  |  |  |  |
| <45 | 9 | 0.11 | **85.12** | 38.92 | 161.59 |
| 45-54 | 14 | 0.33 | **42.42** | 23.19 | 71.17 |
| 55-64 | 16 | 0.92 | **17.47** | 9.98 | 28.36 |
| 65-74 | 20 | 2.60 | **7.68** | 4.69 | 11.86 |
| 75+ | 23 | 4.19 | **5.49** | 3.48 | 8.23 |
| Year of diagnosis |  |  |  |  |  |
| 2000-2004 | 30 | 3.74 | **8.03** | 5.42 | 11.46 |
| 2005-2009 | 25 | 2.70 | **9.26** | 5.99 | 13.67 |
| 2010-2015 | 27 | 1.71 | **15.78** | 10.40 | 22.96 |
| Latency, months |  |  |  |  |  |
| 12-59 | 35 | 3.81 | **9.19** | 6.40 | 12.79 |
| 60-119 | 30 | 2.77 | **10.83** | 7.30 | 15.45 |
| 120+ | 17 | 1.46 | **11.61** | 6.77 | 18.59 |

Observed/expected numbers (O/E) represent the standardized incidence ratios (SIRs), of which all groups are statistically significant (*p* < 0.05). *RGCs* remnant gastric cancers; *FPGC* first primary gastric cancer; *CI* 95% confidence interval.
